# Supplementary material for: Cryo-EM structures of engineered active bc1-cbb3 type CIII2CIV super-complexes and electronic communication between the complexes
Source: Nat Commun. 2021 Feb 10;12:929. doi: 10.1038/s41467-021-21051-4 (PMC7876108; doi:10.1038/s41467-021-21051-4)
Supplement: Supplementary file 1 — Supplementary Information [file 41467_2021_21051_MOESM1_ESM.pdf]

Supplementary Information for
Cryo-EM Structures of Engineered Active bc_1-cbb_3 type $CIII_2CIV$ Super-complexes and Electronic Communication Between the Complexes

Stefan Steimle[1], Trevor van Eeuwen[2], Yavuz Ozturk[1,3], Hee Jong Kim[2], Merav Braitbard[4], Nur Selamoglu[1], Benjamin A. Garcia[5], Dina Schneidman-Duhovny[4], Kenji Murakami[5,]* and Fevzi Daldal[1,]*

[1]Department of Biology, University of Pennsylvania, Philadelphia, PA 19104; [2]Biochemistry and Molecular Biophysics Graduate Group, Perelman School of Medicine, University of Pennsylvania, Philadelphia, PA 19104; [3]Institute of Biochemistry and Molecular Biology, Faculty of Medicine, Albert-Ludwigs University of Freiburg, 79104 Freiburg, Germany; [4]School of Computer Science and Engineering, Institute of Life Sciences, The Hebrew University of Jerusalem, Jerusalem, 9190401, Israel and [5]Department of Biochemistry and Biophysics, Perelman School of Medicine, University of Pennsylvania, Philadelphia, PA 19104

Key words: cytochrome bc_1 or Complex III; Cytochrome cbb_3 oxidase or Complex IV; respiratory super-complex; electron carrier cytochrome c: membrane-anchored cytochrome c_y; soluble cytochrome c_2; *Rhodobacter capsulatus*; respiratory electron transport chain

***Corresponding authors**: Fevzi Daldal: fdaldal@sas.upenn.edu
Phone: +1 215 898-4394

Kenji Murakami: kenjim@pennmedicine.upenn.edu
Phone: +1 215 573-1125

Supplementary Table 1. Strains, plasmids and primers used in this work.

Strain	Description	Phenotype	Reference
E. coli			
HB101	F^-Δ(*gpt-proA*)62 *leuB6 supE44 ara*-14 *galK2 lacY* 1 Δ(*mcrC-mrr*) *rpsL20* (Str^R) *xyl-5 mtl-1recA*13	Str^r	[1]
XL1-Blue	*recA1 endA1 gyrA96 thi-1 hsdr-17 supE44 relA1 lac [F' proAB lacI q ΔM15 Tn10]*	Tet^r	Stratagene
R. capsulatus			
[a]MT1131	*crtD121*Rif^r	Wild Type	[2]
MG1	*ccoP::kan*	Kan^r, Cox^-, $NADI^-$	[3]
YO12	*ccoP::kan Δ*(petABC::Gm)	Kan^r, Cox^-, $NADI^-$, Gm^r, Ps^-	This work
M7G-CBC1	*crtDJ21 cox-410* Δ*(petBC::spe)18*	Spe^r, $Nadi^-$, Myx^r	[4]
F2J-R4	*crtD121 Δ(cycA::kan) Δ(cycY::spe) X4*	cyt c_2^-, cyt c_y^-, X^-, Ps^-, Res^+	[5]
**Plasmid**			
pBSII	pBluescriptII (KS+)	Amp^r	Stratagene
pRK2013	Conjugation helper	Kan^r	[6]
pRK415	Broad host-range vector	Tet^r	[6]
pMTS1	*petABC* on pRK415 derivative with *kan* instead of *tet*	Kan^r	[7]
pOX15	*ccoNOQP* on pRK415	Tet^r	[3]

pYO34	*Δ(petABC::gm)*	Gm[r]	[5]
pYO60	1.13 kb StuI/HindIII fragment with *ccoP-His₈* cloned to StuI/HindIII digested pMTSI to yield *petABC::ccoP-His₈*	Kan[r]	This work
pYO63	*petABC::ccoP-His₈* fusion on 3.9 kb BamHI fragment of pYO60 cloned to pBSII plasmid	Amp[r]	This work
pYO76	*petABC::ccoP-His₈* fusion on 3.9 kb BamHI fragment of pYO60 cloned to pRK415 plasmid	Tet[r]	This work
pYO77	*petABC::ccoP-His₈* fusion linked by 8 AA (**GGSGGGSG)** linker on pBSII plasmid	Amp[r]	This work
pYO78	*petABC::ccoP-His₈* fusion linked by 12 AA (**GGSGGGSGGGSG)** linker on pBSII plasmid	Amp[r]	This work
pYO79	*petABC::ccoP-His₈* fusion linked by 12 AA (**ASIAGGRTASGP)** linker on pBSII plasmid	Amp[r]	This work
pYO80	*petABC::ccoP-His₈* fusion linked by 8 AA (**GGSGGGSG)** linker on pRK415 plasmid	Tet[r]	This work
pYO81	*petABC::ccoP-His₈* fusion linked by 12 AA (**GGSGGGSGGGSG)** linker on pRK415 plasmid	Tet[r]	This work
pYO82	*petABC::ccoP-His₈* fusion linked by 12 AA (**ASIAGGRTASGP)** linker on pRK415 plasmid	Tet[r]	This work
pYO91	619 bp BalI/HinIII fragment containing *cycY-Flag* (without membrane anchor) cloned to BalI/HindIII fragment of pYO63 to yield *petABC::ccoP::cycY-Flag*	Amp[r]	This work
pYO92	4.5 kb KpnI/XbaI fragment of pYO91 containing *petABC::ccoP::cycY-Flag* cloned to pRK415	Tet[r] , cyt bc_1-CcoP-c_y	This work
pYO135	1.2 kb KpnI-BamHI fragment containing the signal sequence of *cycA* and the cyt *c* domain of *cycY* with K19R and H53Y mutations cloned to pRK415	Tet[r], cyt S-c_y K19R H53Y	[5]

Primer name	Sequence		
Fw-ccoP (StuI)	5'-AAGGAGGCGAGGCC***TTG***AGCAAAAAACCGAC-GAC-3'	na	This work
Rv-ccoP (HindIII)	5'-TTGCAACTGAAGCTTAAAGCTATATTGCCGA-TGC-3'	na	This work
[b]Fw-ccoPseq	5'-GGCATCCGCGATCCGCTCG-3'	na	This work
[b]Rv-ccoPseq	5'-CAGATGCGCGGGCATGTTC-3'	na	This work
[c]F-L2	5'-AAAAGGCC***TCG***GGCGGCTCGGGCGGCGGCTCG-GGCAGCAAAAAACCGACGACCAAA-3'	na	This work
[c]F-L3	5'-AAAAGGCC***TCG***GGCGGCTCGGGCGGCGGCTC-GGGCGGCGGCTCGGGCAGCAAAAAACCGACGAC-CAAA-3'	na	This work
[c]F-L4	5'-AAAAGGCC***TCG***GCGTCGATCGCCGGCGGCCG-CACCGCATCGGGCCCGAGCAAAAAACCGACGACCAAA-3'	na	This work
Fw-cp-cy (BalI)	5'-CTTTCTGGCCAGCTCTTTGCGACGCGGCCGGC-3'	na	This work
Rv-cp-cy (BglII+HinIII)	5'-GGGGCAAGCTTGCAAAGATCTGAGGGCAAA-AGG-3'	na	This work

[a]*R. capsulatus* strain MT1131 is derived from SB1003 in multiple steps, as described in [2] ; first, a Ps-deficient mutant (TL1) was obtained using tetracycline suicide, then its *crtD* derivative was constructed by GTA cross to yield MT113, which was used to yield its Ps-proficient derivative via a second GTA cross.

[b]Fw-ccoPseq and Rv-ccoPseq are used for sequencing and the rest of primers for constructions, as described in Methods.

[c]F-L denotes the primers used for inserting the linker sequences (in gray) into the *petC-ccoP* fusion junction. Underlined sequences indicate the restriction sites used for cloning. Italic bold ***TCG*** sequence indicates the stop codon of cyt c_1 changed to a serine codon.

Supplementary Table 2. Identification of protein subunits by mass spectrometry.

Protein	Cofactors	AA	M_r	TMH	PSM	uniqPep	Identified Peptides	X_{corr}	z
**cyt c_1**	heme c_1	258	28	1	60	9	AGFSGPAGSGM*NQLFK	4.71	2
(in c_1-CcoP)							ETDMFPTR	1.68	1
							EYAAGLDTIIDK	3.58	2
							EYAAGLDTIIDKDSGEER	5.00	2
							EYAAGLDTIIDKDSGEERDR	3.24	3
							TFQIGGVPDTCK	3.50	2
							TFQIGGVPDTCKDAAGVK	3.86	3
							TLADDGGPQLDPTFVR	4.50	2
							VGDGM*GPDLSVM*AK	3.94	2
**CcoP**	heme c_{p1}	297	32	1	31	5	ATAGQQVFADNC*VSC*HGEDAK	3.73	3
(in c_1-CcoP)	heme c_{p2}						AVEDKLVATDLTAIAADPELVTYTR	3.94	3
							EVQTTGHSWDGIEELNTPLPR	5.42	2
							KEVQTTGHSWDGIEELNTPLPR	5.31	3
							LVATDLTAIAADPELVTYTR	6.07	3
**CcoN**	heme b	532	59	12	16	3	DEYFDGVIR	2.69	2
	heme b_3						EVDAQGFLVNGFADTVGAK	5.38	2
							TM*GDAKPSKDEYFDGVIR	3.83	3
**cyt *b***	heme b_H	437	49	8	20	5	ADAEKDTLPFWPYFVIK	3.92	3
	heme b_L						DTLPFWPYFVIK	3.28	2
							DVNGGWAMR	3.39	2
							GLYYGSYK	2.05	1
							LPIVGLVYDTIM*IPTPK	5.40	2
**CcoO**	heme c_o	242	27	1	23	4	ADFVAQADPNADSATLVANYGEK	5.45	2
							VEGM*RPYTPLELTGR	3.64	2/3
							VSTDALVGVPYSAEM*IAAAK	4.61	2/3
							YGHYSLAAESM*YDHPFQWGSK	4.61	3

**FeS protein**	2Fe2S	191	20	1	9	3	IRKGPAPRNLDIPVAAFVDETTIK	2.73	3
(Rieske)							NLDIPVAAFVDETTIK	4.37	2
							SGDFGGWFC*PC*HGSHYDSAGR	3.74	3
**cyt c_1**	heme c_1	258	28	1	27	12	TLADDGGPQLDPTFVR	4.59	2
(in c_1-CcoP-c_y)							AGFSGPAGSGM*NQLFK	4.38	2
							EYAAGLDTIIDKDSGEER	3.52	2
							EYAAGLDTIIDKDSGEERDRK	3.34	4
							VGDGM*GPDLSVM*AK	3.26	2
							TFQIGGVPDTC*K	3.00	2
							EYAAGLDTIIDKDSGEERDR	2.94	4
							EYAAGLDTIIDK	2.68	2
							DRKETDMFPTR	2.66	3
							KETDMFPTR	2.32	2
							ETDM*FPTR	2.21	2
							YDQAQLR	2.10	2
**CcoP**	heme c_{p1}	297	32	1	16	5	ATAGQQVFADNC*VSC*HGEDAK	3.45	3
(in c_1-CcoP-c_y)	heme c_{p2}						ADVEKDIAK	3.13	2
							ISGQPADEAR	2.82	2
							NAGAAVFR	2.56	2
							LSEAQIR	2.08	2
**cyt c_y**	heme c_y	199	21	1	14	7	M*SFVGLPEAADR	3.70	2
(in c_1-CcoP-c_y)							ATATVEGFKYSTAM*K	3.21	3
							ANVIAYLNTLPR	3.03	2
							ALLPSVDEAAM*PAK	2.81	2
							LDIYLVSPK	2.41	2
							NHVGNWTPER	2.27	2
							ATATVEGFK	2.20	2

All proteins were identified using the bipartite SC preparation, except the c_1-CcoP-c_y fusion peptide, which was identified using the tripartite SC. The numbers of peptide spectral matches (PSM) and unique peptides (uniqPep) are given for each protein. The

sequences of the identified peptides are listed along with their X-correlation (X_{corr}) and charges (z) [8]. Some peptides were identified with and without various modifications; residues that were found in the modified version are marked with an asterisk; modifications were oxidation in the case of methionine (M*) and carbamidomethylation in the case of cysteine (C*). Additional information provided for each protein subunit includes the number of amino acid residues (AA), molecular mass (M_r) in kDa, number of transmembrane helices (TMH) and the cofactors content.

Supplementary Table 3. Cryo-EM data collection, refinement and validation statistics

	#1 SC-1A (EMDB-22228) (PDB 6XKX)	#2 SC-1B (EMDB-22230) (PDB 6XKZ)	#3 SC-2A (EMDB-22227) (PDB 6XKW)	#4 $CIII_2$ (EMDB-22189) (PDB 6XI0)	#5 $CIII_2$-cc (EMDB-22224) (PDB 6XKT)	#6 $CIII_2$-bc (EMDB-22225) (PDB 6XKU)	#7 $CIII_2$-bb (EMDB-22226) (PDB 6XKV)
**Data collection and processing**							
Magnification	105,000	105,000	64,000	64,000	64,000	64,000	64,000
Voltage (kV)	300	300	300	300	300	300	300
Electron exposure (e–/Å^2)	40	40	40	40	40	40	40
Defocus range (μm)	0.4 - 4.0	0.4 - 4.0	0.4 - 4.0	0.4 - 4.0	0.4 - 4.0	0.4 - 4.0	0.4 - 4.0
Pixel size (Å)	1.32	1.32	1.36	1.36	1.36	1.36	1.36
Symmetry imposed	none	none	none	C2	C2	none	C2
Initial particle images (no.)	560,860	623,077	716,907	733,210	733,210	733,210	733,210
Final particle images (no.)	61,934	87,026	14,978	37,997	35,069	26,254	37,710
Map resolution (Å) FSC threshold 0.143 (0.5)	6.09 (7.14)	7.20 (8.85)	5.18 (7.35)	3.30 (3.70)	3.75 (4.15)	4.18 (4.72)	3.47 (3.89)
Map resolution range (Å)	4.5 - 9.0	4.8 - 9.0	4.3 - 8.0	3.0 - 4.0	3.25 - 4.5	3.65 - 5.0	3.0 - 4.0
**Refinement**							
Initial model used (PDB code)	6XI0 + 3MK7*	6XI0 + 3MK7*	6XI0 + 3MK7*	1ZRT	6XI0	6XI0	6XI0
Model resolution (Å) FSC threshold 0.5	7.87	9.14	7.32	3.41	3.88	4.29	3.65
Model resolution range (Å)	N/A	N/A	N/A	N/A	N/A	N/A	N/A
Map sharpening B factor (Å^2)	-184	-684	-87	-33	-71	-50	-54
Model composition							
Non-hydrogen atoms	20,409	20,409	20,409	13,079	13,079	13,079	13,079
Protein residues	2,593	2,593	2,593	1,678	1,678	1,678	1,678
Ligands	14	14	14	8	8	8	8
B factors (Å^2)							
Protein	67.41	67.41	69.91	44.55	44.47	44.51	44.55
Ligand	72.66	72.66	72.66	52.01	53.03	52.52	52.01
R.m.s. deviations							
Bond lengths (Å)	0.012	0.012	0.012	0.007	0.008	0.008	0.007
Bond angles (°)	1.601	1.601	1.590	0.971	1.174	1.182	0.976
Validation							
MolProbity score	3.10	3.12	3.09	2.23	2.28	2.29	2.22
Clashscore	58.95	59.15	59.42	22.30	24.97	24.22	22.03
Poor rotamers (%)	2.12	2.12	2.07	0.08	0.08	0.16	0.08
Ramachandran plot							
Favored (%)	87.61	87.57	87.70	83.23	83.11	83.17	83.17
Allowed (%)	11.88	11.92	11.76	16.34	16.47	16.47	16.41
Disallowed (%)	0.51	0.51	0.54	0.42	0.42	0.36	0.42

*a homology model was created from 3MK7

Supplementary Table 4. Cross-links identified in samples treated with DMTMM or DSBU.

Protein 1	Protein 2	Peptide 1	Peptide 2	Abs Pos 1	Abs Pos 2	MeroX Score	Dist [Å]
**Intra-Subunit Cross Links with DMTMM**							
FeS protein	FeS protein	GKPVFIR	DIELAR	70	81	81	24.6
FeS protein	FeS protein	DTSAENANKPGAEATDENR	DEKDIELAR	107	80	72	22.3
FeS protein	FeS protein	DTSAENANKPGAEATDENR	KGPAPR	107	168	68	19.7
FeS protein	FeS protein	SGDFGGWFC*PC*HGSHYDSAGR	KGPAPR	147	168	91	8.5
cyt c_1	cyt c_1	NSNVPDHAFSFEGIFGK	KETDMFPTR	2	84	93	@
cyt c_1	cyt c_1	NSNVPDHAFSFEGIFGK	VGDGMGPDLSVM*AK	2	99	72	@
cyt c_1	cyt c_1	EYAAGLDTIIDKDSGEER	#NSNVPDHAFSFEGIFGK	63	-1	90	@
cyt c_1	cyt c_1	EYAAGLDTIIDKDSGEER	ETDMFPTR	74	84	57	15.1
cyt c_1	cyt c_1	DRKETDMFPTR	DRKETDMFPTR	81	83	48	††6.0
cyt c_1	cyt c_1	VGDGMGPDLSVM*AKAR	KETDM*FPTR	105	86	47	23.1
CcoP	CcoP	EVQTTGHSWDGIEELNTPLPR	KPTTK	23	4	76	@
CcoP	CcoP	ADVEKDIAKFAEM*NK	ADVEKDIAKFAEMNK	76	75	48	††3.8
CcoP	CcoP	FAEMNKAVEDK	ADVEKDIAK	85	74	74	14.8
cyt c_2	cyt c_2	GAKTGPNLYGVVGR	TAGTYPEFK	32	50	73	9.9
cyt c_2	cyt c_2	YKDSIVALGASGFAWTEEDIATYVK	DPGAFLK	54	78	60	17.2
cyt c_2	cyt c_2	YKDSIVALGASGFAWTEEDIATYVK	AKTGMAFK	55	93	58	8.7
cyt c_2	cyt c_2	YKDSIVALGASGFAWTEEDIATYVK	TGMAFKLAK	70	99	60	15.8
cyt c_2	cyt c_2	YKDSIVALGASGFAWTEEDIATYVK	DPGAFLKEKLDDKK	71	84	60	14.0
cyt c_2	cyt c_2	DPGAFLKEK	EKLDDK	84	85	71	††3.8
cyt c_2	cyt c_2	EKLDDKK	AKTGMAFK	85	93	82	11.2
cyt c_2	cyt c_2	TGMAFKLAK	DPGAFLK	99	78	89	6.1
cyt c_2	cyt c_2	TGM*AFKLAK	EKLDDKK	99	85	58	15.9
cyt c_2	cyt c_2	LAKGGEDVAAYLASVVK	EKLDDKK	102	85	78	20.0
cyt c_2	cyt c_2	GGEDVAAYLASVVK	TGM*AFKLAK	106	99	60	15.2

**Inter-Subunit Cross Links with DMTMM (Intra- and Inter-Complex)**							
Cyt c_1	FeS protein	VG**D**GMGPDLSVMAK	**K**GPAPR	94	168	80	
CcoO	CcoP	V**E**GM*RPYTPLELTGR	ADVE**K**DIAK	47	75	62	26.2
CcoO	CcoP	V**E**GM*RPYTPLELTGR	FAEMN**K**AVEDK	47	85	36	32.1
FeS protein	CcoP	**D**TSAENANKPGAEATDENR	GGVMPSWSWAADGA**K**PR	95	275	51	
FeS protein	CcoP	DTSAENANKPGA**E**ATDENR	FAEMN**K**AVEDK	107	85	66	
**Cyt c_2 and cyt c_y Cross Links with DMTMM**							
cyt c_1	cyt c_2	**E**YAAGLDTIIDKDSGEER	A**K**TGMAFK	63	93	94	
cyt c_1	cyt c_2	VG**D**GMGPDLSVMAK	A**K**TGMAFK	94	93	90	
cyt c_2	cyt c_1	LA**K**GGEDVAAYLASVVK	**E**YAAGLDTIIDK	102	63	51	
CcoP	cyt c_2	LVAT**D**LTAIAADPELVTYTR	LAKGGEDVAAYLASVV**K**	95	116	74	
CcoO	cyt c_2	V**E**GM*RPYTPLELTGR	A**K**TGMAFK	47	93	73	
CcoO	cyt c_2	V**E**GM*RPYTPLELTGR	TGM*AF**K**LAK	47	99	79	
CcoO	cyt c_2	V**E**GM*RPYTPLELTGR	LA**K**GGEDVAAYLASVVK	47	102	60	
CcoO	cyt c_2	ADFVAQADPNA**D**SATLVANYGEK	A**K**TGMAFK	191	93	59	
CcoO	cyt c_2	ADFVAQADPNA**D**SATLVANYGEK	LA**K**GGEDVAAYLASVVK	191	102	73	
CcoO	cyt c_2	ADFVAQADPNADSATLVANYG**E**K	GE**K**EFNK	201	8	72	
CcoO	cyt c_2	ADFVAQADPNADSATLVANYG**E**K	GA**K**TGPNLYGVVGR	201	32	73	
CcoO	cyt c_2	ADFVAQADPNADSATLVANYG**E**K	DPGAFL**K**EK	201	84	63	
FeS protein	cyt c_y	DTSAENANKPGA**E**ATDENR	ATATVEGF**K**YSTAMK	107	143	65	
Cyt c_y	Cyt c_1	ATATVEGF**K**YSTAMK	VG**D**GMGPDLSVMAK	143	94	74	
Cyt c_1	Cyt c_y	**#**NSNVPDHAFSFEGIFGK	ALLPSV**D**EAAM*PAK	-1	54	77	
Cyt c_1	Cyt c_y	E**Y**AAGLDTIIDKDSGEER	ATATV**E**GFKYSTAMK	64	140	69	
Cyt c_1	Cyt c_y	EYAAGLDTIIDKDSG**E**ER	ATATVEGF**K**YSTAMK	78	143	23	
Cyt c_1	Cyt c_y	EYAAGLDTIIDKDSG**E**ER	AEVPGT**K**MSFVGLPEAADR	78	175	62	
**Intra-Subunit Cross Links with DSBU**							
FeS protein	FeS protein	DE**K**DIELAR	G**K**PVFIR	80	70	64	28.2
FeS protein	FeS protein	DTSAENAN**K**PGAEATDENR	RDE**K**DIELAR	103	80	36	26.2
FeS protein	FeS protein	SVPLGALRDTSAENAN**K**PGAEATDENR	**K**GPAPR	103	168	18	26.1

cyt c_1	cyt c_1	EYAAGLDTIID**K**DSGEER	**K**ETDMFPTR	74	83	78	13.1
cyt c_1	cyt c_1	EYAAGLDTIID**K**DSGEER	VGDGMGPDLSVMA**K**AR	74	105	80	29.9
cyt c_1	cyt c_1	VGDGM*GPDLSVMA**K**AR	**K**ETDMFPTR	105	83	75	28.0
cyt c_1	cyt c_1	TFQIGGVPDTC***K**DAAGVK	VGDGMGPDLSVMA**K**AR	168	105	73	29.8
CcoP	CcoP	FAEMN**K**AVEDK	ADVE**K**DIAK	85	75	95	14.2
CcoP	CcoP	AVED**K**LVATDLTAIAADPELVTYTR	ADVEKDIA**K**FAEMNK	90	79	62	17.7
CcoP	CcoP	GGVMPSWSWAADGA**K**PR	FAEMN**K**AVEDK	275	85	74	10.0
cyt c_y	cyt c_y	AEVPGT**K**MSFVGLPEAADR	ATATVEGF**K**YSTAMK	175	143	67	9.8
**Inter-Subunit Cross Links with DSBU (Intra-Complex)**							
FeS protein	cyt c_1	RDE**K**DIELAR	**K**ETDMFPTR	80	83	34	
FeS protein	cyt c_1	DTSAENAN**K**PGAEATDENR	VGDGMGPDLSVMA**K**AR	103	105	35	
cyt c_1	FeS protein	EYAAGLDTIID**K**DSGEER	G**K**PVFIR	74	70	81	
cyt c_1	FeS protein	EYAAGLDTIID**K**DSGEERDRK	**K**GPAPR	74	168	66	
cyt c_1	FeS protein	DR**K**ETDMFPTR	RDE**K**DIELAR	83	80	47	
cyt c_1	FeS protein	VGDGMGPDLSVMA**K**AR	RRDE**K**DIELAR	105	80	49	
cyt c_1	FeS protein	VGDGMGPDLSVMA**K**AR	**K**GPAPR	105	168	71	
cyt c_1	FeS protein	TFQIGGVPDTC***K**DAAGVK	RDE**K**DIELAR	168	80	36	
cyt c_1	FeS protein	TFQIGGVPDTC***K**DAAGVK	**K**GPAPR	168	168	77	
**Inter-Complex Cross Links with DSBU (Intra-Supercomplex)**							
cyt c_1	CcoP	DR**K**ETDMFPTR	FAEMN**K**AVEDK	83	85	29	51.6
cyt c_1	CcoP	DAAGV**K**ITHGSWAR	ADVE**K**DIAK	174	75	73	16.1
CcoP	cyt c_1	ADVE**K**DIAKFAEM*NK	**K**ETDMFPTR	75	83	55	56.6
CcoP	cyt c_1	ADVEKDIA**K**FAEMNK	VGDGMGPDLSVMA**K**AR	79	105	60	28.0
CcoP	cyt c_1	FAEMN**K**AVEDK	**K**ETDMFPTR	85	83	70	51.6
CcoP	cyt c_1	GGVMPSWSWAADGA**K**PR	**K**ETDMFPTR	275	83	56	58.2
CcoP	FeS protein	ADVE**K**DIAKFAEM*NK	**K**GPAPR	75	168	55	
CcoP	FeS protein	DIA**K**FAEM*NK	**K**GPAPR	79	168	34	
CcoP	FeS protein	FAEMN**K**AVEDK	**K**GPAPR	85	168	56	
CcoP	FeS protein	GGVMPSWSWAADGA**K**PR	**K**GPAPR	275	168	51	

Cyt c_y Cross Links with DSBU							
cyt c_y	FeS protein	IDG**K**NAVGPHLNGVIGR	**K**GPAPR	121	168	47	
cyt c_y	FeS protein	ATATVEGF**K**YSTAMK	RRDE**K**DIELAR	143	80	23	
cyt c_y	FeS protein	LDIYLVSP**K**AEVPGTK	**K**GPAPR	168	168	32	
cyt c_y	FeS protein	AEVPGT**K**M*SFVGLPEAADR	**K**GPAPR	175	168	27	

Cross-linked amino acids are indicated in bold red letters. The absolute position (Abs Pos) numbering of the cross links corresponds to the amino acid sequences of *R. capsulatus* proteins in mature proteins, as in PDB: 6XI0. -1 refers to linkage to the N-ter NH_2 of the protein. Oxidized Met and alkylated Cys residues are noted with *, and N-terminal linked amino acids are noted with #. Uniprot IDs for the proteins are: FeS protein, D5ANZ2; cyt c_1, D5ANZ4; CcoP, D5ARP7; CcoO, D5ARP5; cyt c_2, P00094 and cyt c_y, Q05389. The high level confidence cross-linked peptides listed are those identified by a minimum of two search engines: MeroX [9] and FindXL [10] in the case of DMTMM, and MeroX [9] and MassAI (http://www.massai.dk) in the case of DSBU. As detailed in Methods, the FDR confidence levels from the MeroX search results were filtered to <1%, and the MeroX scores are included in the table for reference. Other search details and parameters are given in Methods. Using the structural model SC-2A, the distances that separate cognate XL pairs within the nonflexible portions of SC (*i.e.*, excluding FeS, cyt c_2, cyt c_y) are listed under column Dist [Å], and the XLs within the same peptide are marked with ††. @ indicates the distances between the N-ter or C-ter of non modeled residues of cyt c_1 or CcoP that are not resolved. The distances that are not indicated involve the flexible FeS-ED domain or the docking models of cyt c_2 and cyt c_y.

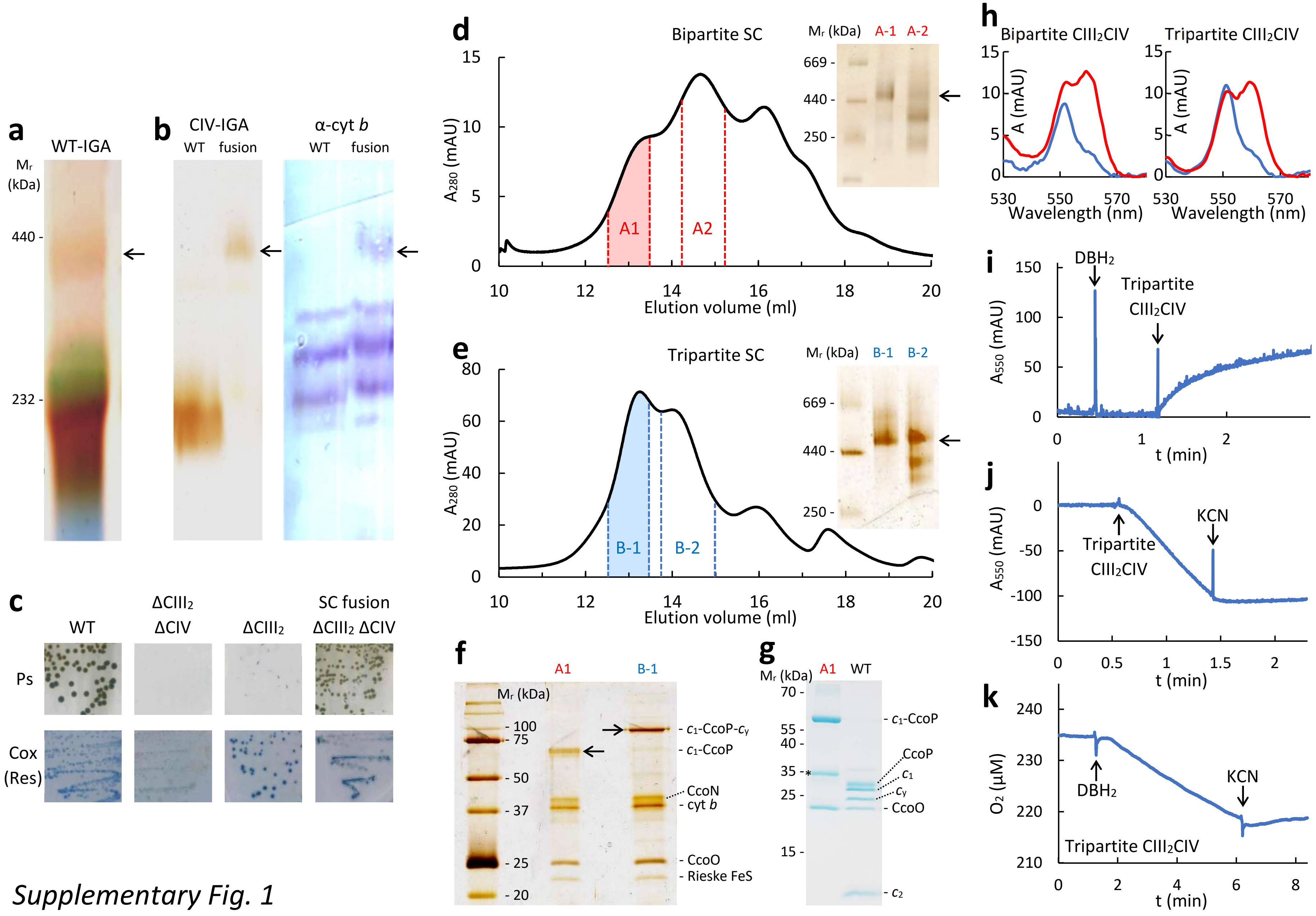

Supplementary Fig. 1

Supplementary Figure 1. Characterization of *R. capsulatus* wild type and fusion SCs. a. DDM-dispersed membranes from *R. capsulatus* wild-type strain MT1131 were separated in 4-13% BN-PAGE and the gel was overstained for CIV-in-gel activity (SI Methods) to detect the ~440 kDa entity. Under the conditions used, CIV runs as a diffuse ~232 kDa band, just below the "green band" of bacteriochlorophyll-complexes. **b.** DDM-dispersed membranes from *R. capsulatus* wild-type strain MT1131(WT), and its mutant derivative YO12, lacking both $CIII_2$ and CIV but carrying plasmid pYO76 and expressing the bipartite SC (fusion), were separated in 4-16% BN-PAGE. One part of the gel was stained for CIV-in-gel activity (CIV-IGA, left), and the other part blotted onto a PVDF membrane and treated with *R. capsulatus* cyt *b* specific antibodies (α-cyt *b*, right), detecting multiple forms of $CIII_2$ running between ~250-300 kDa M_r. The size and position of HMW markers (GE Healthcare) are indicated, and arrows points out the SCs of ~440 kDa M_r. The intact SCs are not readily visible in a wild type strain due to their low amounts and fragile nature. **c**. Growth properties of *R. capsulatus* strain pYO76/YO12, lacking $CIII_2$ and CIV but carrying a bipartite SC (fusion). The top and bottom rows show, respectively, the photosynthetic growth (Ps) and the *in vivo* Cox activity (NADI-staining, Methods) observed under respiratory (Res) growth conditions of colonies from wild type (WT), YO12 lacking both $CIII_2$ and CIV (Δ$CIII_2$ ΔCIV), YO12 carrying a plasmid (pOX15) containing only CIV (*i.e.*, Δ$CIII_2$); and YO12 carrying a plasmid (pYO76) expressing the bipartite fusion SC (SC fusion Δ$CIII_2$ ΔCIV) (**Supplementary Table 1**). **d** and **e.** SEC elution profiles of the bipartite SC (**d**) and tripartite SC (**e**). In each case, the fractions 1 and 2 were analyzed by 4-16% Native PAGE (insets) and silver staining, and only the fractions A-1 and B-1 were used for cryo-EM studies. The bands at ~450/480 kDa corresponding to bipartite and tripartite SCs are indicated by arrows. **f.** Fractions A-1 and B-1 were separated by SDS-PAGE, silver stained, and protein bands identified by mass spectrometry. The fused bipartite c_1-CcoP (A-1) and tripartite c_1-

CcoP-c_y (B-1) subunits are indicated by arrows. Note the absence of c_1 and CcoP subunits in both cases. **g**. Peak A-1 and DDM-dispersed membranes from wild type *R. capsulatus* (WT) were analyzed by SDS-PAGE/TMBZ to reveal the covalently attached heme cofactors. The tripartite construct (B-1) is virtually identical to A-1, except that the c_1-CcoP band is replaced by c_1-CcoP-c_y. All *c*-type cyts are labeled, and the band indicated by * corresponds to a proteolytic cleavage product of the c_1-CcoP fusion subunit. **h.** Redox difference spectra of the bipartite (Bipartite $CIII_2CIV$, left) and tripartite (Tripartite $CIII_2CIV$, right) SCs. Ascorbate-reduced *minus* ferricyanide-oxidized visible spectra (blue) revealing the *c*-type cyts at 550 nm, and dithionite-reduced *minus* ferricyanide-oxidized spectra (red) revealing both *c*-type and *b*-type cyts at 550 and 560 nm, respectively, are shown. **i** and **j.** DBH_2:cyt *c* reductase (7.2 +/-2.1 µmol/mg protein/min) and cyt c:O_2 reductase (2.9 +/-0.7 µmol/mg protein/min) activities of tripartite SCs monitored at 550 nm (Methods). **k.** DBH_2 dependent O_2 consumption activity (0.143 +/-0.025 µmol of O_2 consumed/mg of protein/min) of tripartite SCs. Arrows indicate the additions of DBH_2, KCN and purified tripartite SCs as appropriate. Similar DBH_2:cyt *c* reductase (12.4 +/-1.8 µmol/mg of protein/min) and cyt c:O_2 reductase (0.46 +/-0.14 µmol/mg of protein/min) were also observed with the bipartite SCs, which shows no O_2 consumption activity in the absence of an external electron carrier cyt *c* (not shown). Source Data for this figure are provided as a Source Data file. All experiments were repeated at least three times with independent samples and similar results.

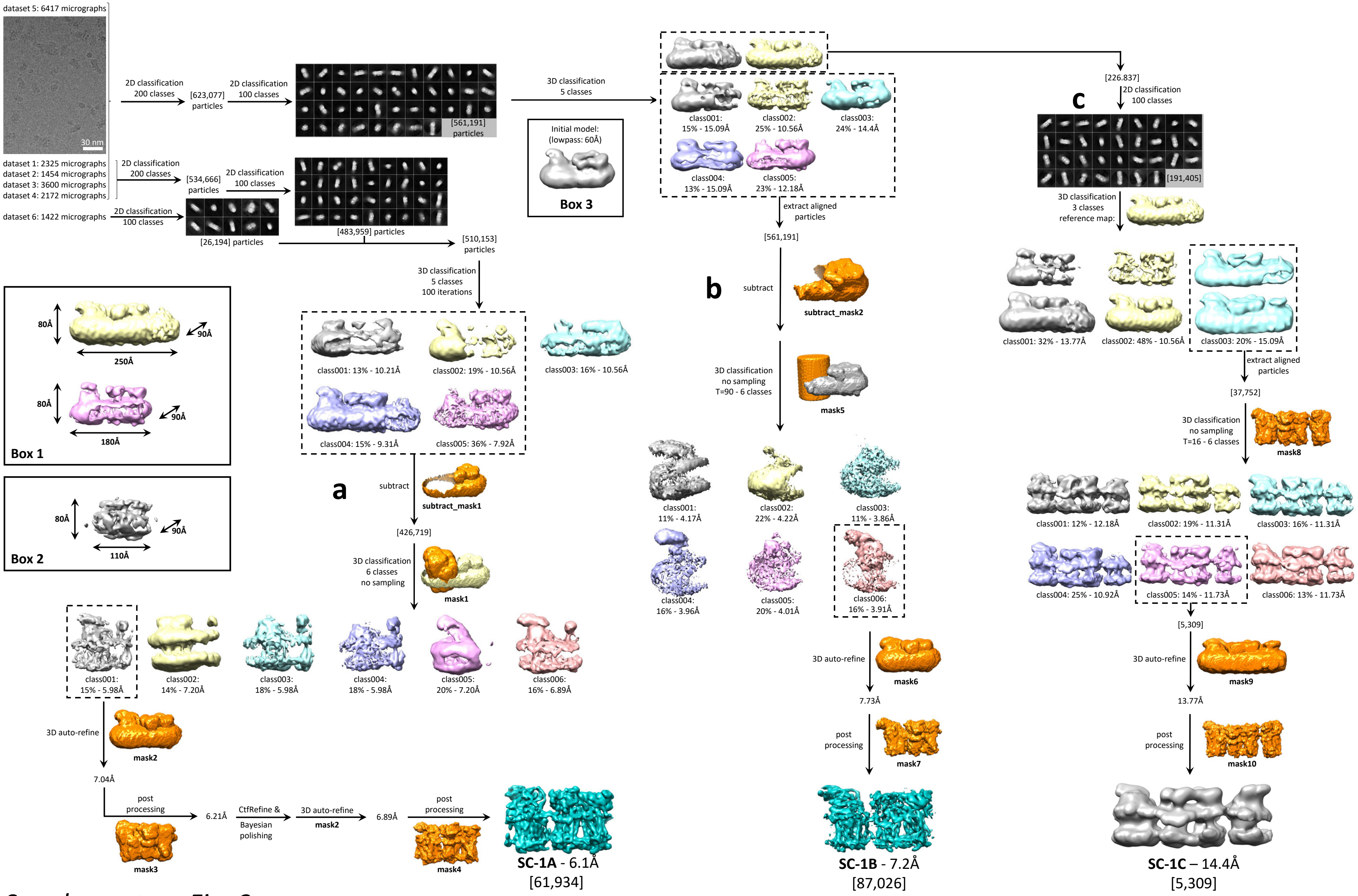

Supplementary Fig. 2

Supplementary Figure 2. Classification tree for tripartite SCs. Six datasets (dataset-1 to 6) were collected. One representative micrograph (out of 17,390) is shown on the top left corner with a white 30 nm scale bar. The datasets were processed by Relion 3.0 as appropriate. Box 1 shows the dimensions of major particle species identified in this sample, Box 2 shows an additional species observed in the bipartite sample. For simplicity, only the analyses leading to the best version of relevant maps are shown. In all cases the subclasses obtained in 3D classifications are shown in different colors, and both the class distribution (%) and nominal resolution (Å) are indicated. The masks are shown in orange (not scaled to maps) and the final maps deposited to EMDB in turquoise. After one or two rounds of 2D classification, best 2D class averages were retained (~500,000 particles) and classified into three to five 3D classes, using an initial model obtained from dataset 1 and lowpass-filtered to 60 Å (Box 3). **a.** Analysis of the datasets 1-4 and 6. All classes showing strong density for *cbb*$_3$-type CIV were retained and subjected to focused classification. A soft mask was wrapped around the CIV portion of SCs, information outside the mask was subtracted from the particle images, and particles inside the mask (mask1 in orange with subtract_mask1 in yellow) were sorted into 6 classes. The class showing a complete map of CIV and the highest level of detail was retained and subjected to 3D auto-refinement and post processing. The last two steps were repeated after CTF refinement and Bayesian polishing. The final map (SC-1A) contained 61,934 particles, and had a nominal resolution of 6.09 Å. **b.** A similar strategy was used for the analysis of datasets 1-5, except that a cylindrical mask around the CIV portion was used, and the CTF refinement and Bayesian polishing are omitted, as they did not improve the resolution. The final map (SC-1B) showed a different conformation of CIV relative to CIII$_2$ as compared to map SC-1A, contained 87,026 particles and had a nominal resolution of 7.2 Å. **c**. Two classes showing density corresponding to a second copy of CIV associated with CIII$_2$ were selected, and the particles subjected to another round of 2D

classification. The class averages showing the highest level of details were retained, and the particles classified into three 3D classes, of which the class showing the highest occupancy of the second copy of CIV associated with $CIII_2$ was sorted into 6 subclasses using a solvent mask. The classes with the strongest density for the second copy of CIV were subjected to refinement and post processing, and one such class (SC-1C, in gray) is shown. Due to low number of particles in each class (~5,000) these maps could not be refined to high resolution.

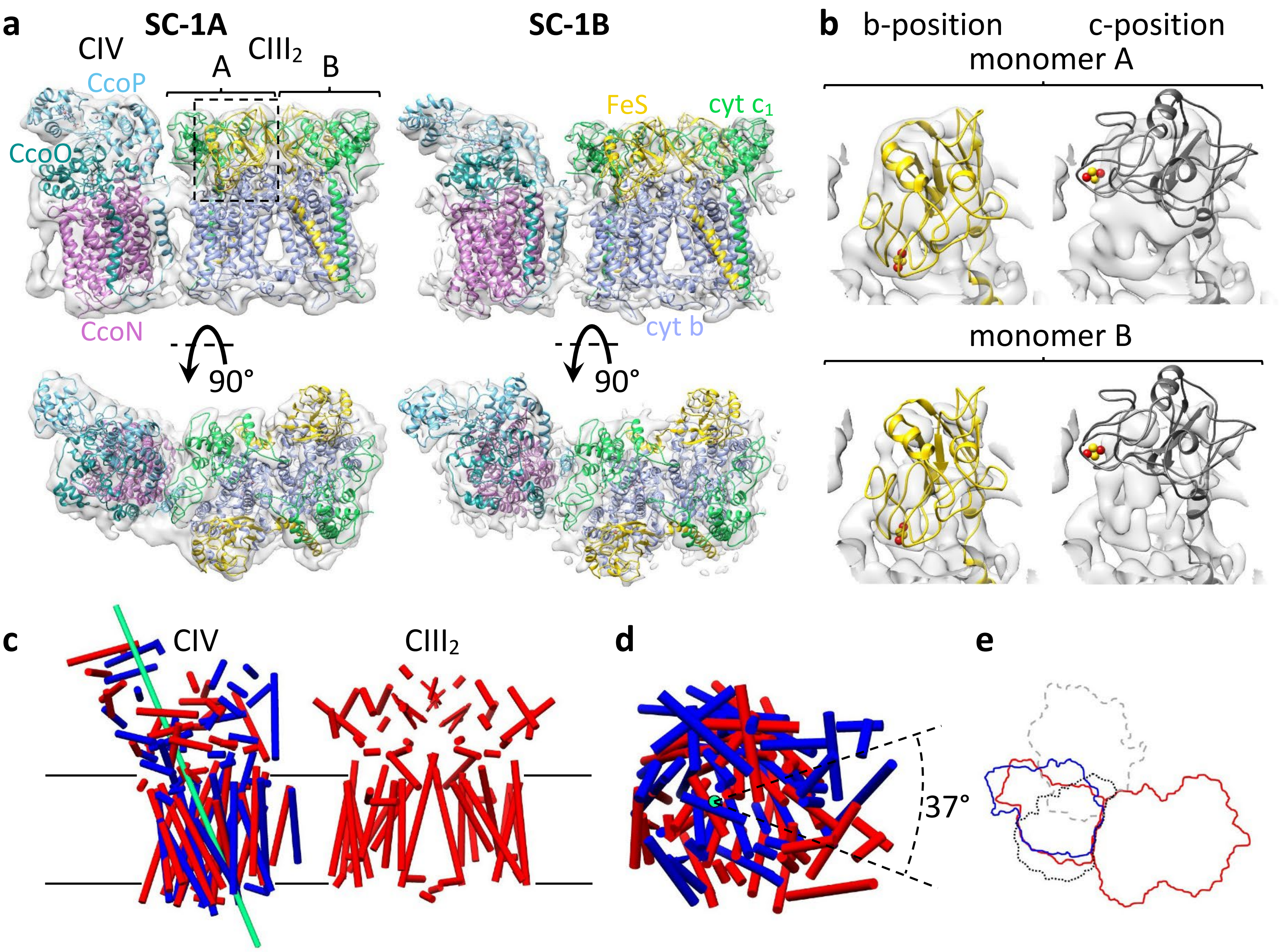

Supplementary Fig. 3

Supplementary Figure 3. Different orientations of CIV relative to $CIII_2$ in tripartite SCs. a. The $CIII_2$ structure (PDB: 6XI0) and the CIV homology model were fitted into the density maps SC-1A and SC-1B; side and top views are shown. Subunits are colored and labeled as in **Fig. 2a** and the monomers A and B of $CIII_2$ are indicated. The region corresponding to panel **b** is highlighted with a dashed box in monomer A. **b.** Fitting of the FeS-ED in both $CIII_2$ monomers A and B of map SC-1A (EMD-22228). $CIII_2$ models with their FeS-EDs in b- (PDB: 6XI0, yellow) or in c-positions (PDB: 6XKT, grey) were fitted into SC-1A. Although the resolution of the FeS-EDs is low, clearly, they are located closer to the b-position in SC-1A. The map is shown at the same sigma level in all panels for direct comparison, and the lover occupancy in monomer B is noted. A similar situation was observed in SC-1B. **c.** The CIV homology and $CIII_2$ (PDB: 6XI0) models were fitted into SC1-A and SC-1B maps and the $CIII_2$ portions were superimposed to visualize the different orientations of CIV in the tripartite SCs. Only the helical and beta-sheets regions of the proteins are depicted as cylinders and arrows, respectively, with the coils omitted for clarity, and the lipid bilayer is indicated by horizontal lines. Red represents $CIII_2$ and CIV fitted into SC-1A and blue represents CIV fitted into SC-1B. The rotation/translation axis (green) transforming one orientation of CIV into the other one was determined using DynDom3D (dyndom.cmp.uea.ac.uk) [11] with default parameters. **d**. Top view of only the CIV portions of SCs shown along the rotation/translation axis (green dot) and the 37° rotation between the orientations of CIV (colored as in **c**). **e**. Top view (perpendicular to the membrane plane) of the contour maps of tripartite $CIII_2CIV$. The surface contours of $CIII_2$ (right) and CIV (left) in map SC-1A (red), and the different orientation of CIV relative to $CIII_2$ in map SC-1B (blue) are shown. For comparison, the orientations of CIV with respect to $CIII_2$ in *S. cerevisiae* $CIII_2CIV$ (light gray, dashed line, PDB: 6GIQ) and in mycobacterial $CIII_2CIV_2$ (black dotted line, PDB: 6HWH) are shown after superimposition of the cyt *b* structures of all $CIII_2$.

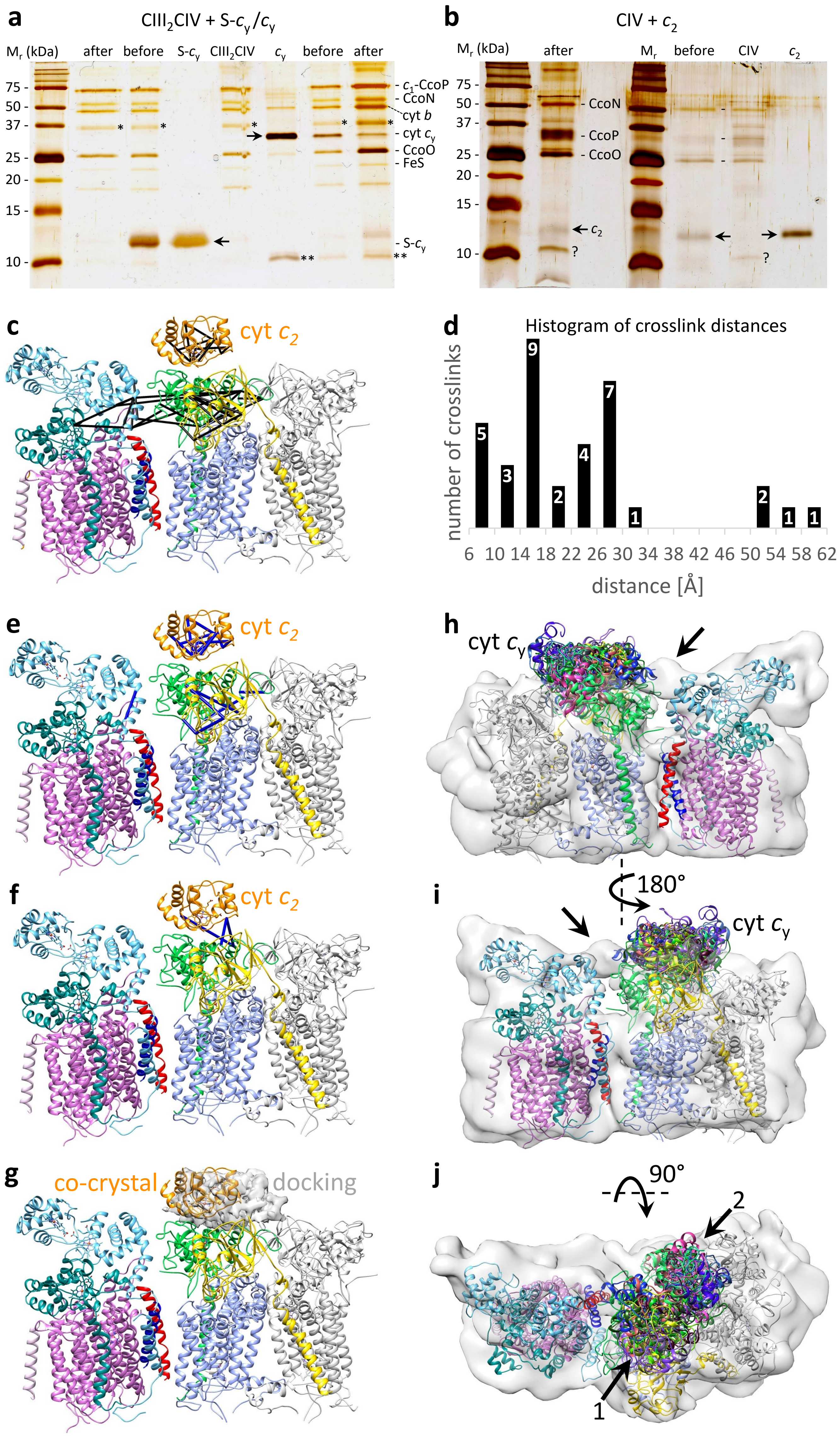

Supplementary Fig. 4

Supplementary Figure 4. Binding of the cyt c_y, cyt S-c_y and cyt c_2 to bipartite SC, and XL-MS guided docking of cyt c_2 and cyt c_y to $CIII_2$. a. Binding of cyt domain of c_y (S-c_y, left) and full-length native cyt c_y (c_y, right) to bipartite SC ($CIII_2CIV$). Purified SCs were mixed with cyt S-c_y or cyt c_y and subjected to SEC. Samples before and after SEC were separated on 18% SDS-PAGE and silver stained. Only cyt c_y, but not S-c_y, co-eluted with bipartite $CIII_2CIV$. Purified samples of cyt c_y, cyt S-c_y (indicated by arrows) and $CIII_2CIV$ are shown for comparison. * and ** indicate the degradation product(s) of c_1-CcoP subunit of bipartite SCs and of cyt c_y, respectively. **b.** Binding of cyt c_2 to cbb_3-type CIV. These proteins were mixed, subjected to SEC, followed by SDS-PAGE and silver staining as in **a**. Non-mixed samples are shown for comparison. CIV was partially purified, its subunits are indicated by dashes, and the nature of the other bands, including that of ~10 kDa indicated by ?, are not known. The cyt c_2 (indicated by an arrow) is coeluted with CIV. Due to limited availability of materials and because results were expected to be reproducible, the experiments shown in **a** and **b** were not repeated. **c.** Overview of crosslinks obtained using XL-MS (**Supplementary Table 4**), shown within the tripartite SC structure SC-2A (colored as in **Fig. 2**, except that $CIII_2$ monomer B is shown in grey). Not included are crosslinks to and from cyt c_2 and cyt c_y which were used as distance restraints for docking and lead to clusters of possible binding positions. Both c_2 and c_y have binding sites on $CIII_2$ and on CIV, but their exact location remains unknown, thus it is impossible to list precise distances for these XLs. Likewise, XLs to and from the mobile Rieske FeS-ED protein are excluded since which of the possible conformations are crosslinked is unknown. **d.** Histogram of distances between the crosslinked positions shown in panel **c**. Four crosslinks in the range of 50-60 Å can be explained by the relative flexibility of $CIII_2$ vs CIV as indicated by the 2 conformers SC-1A and SC-1B (**Supplementary Fig. 3**). **e.** Intra-subunit DMTMM crosslinks are shown. Cyt c_2 (orange, PDB:

1C2N) is shown on monomer A of *R. capsulatus* $CIII_2$, at the position defined by alignment with the yeast iso-1 cyt *c*-$CIII_2$ co-crystal structure (PDB: 3CX5). **f.** XLs between cyt c_2 and $CIII_2$ are shown. Like in **e**, all XLs are within the allowed maximum distance (30Å for DMTMM). **g**. The binding site of *R. capsulatus* cyt c_2-$CIII_2$ defined by alignment with yeast co-crystal structure is compared with the binding regions defined by docking cyt c_2 to $CIII_2$ using Patchdock guided by the DMTMM XLs as distance restraints. The gray transparent volume, encompassing the binding regions of all cyt c_2 models docked on monomer A of $CIII_2$, was generated by choosing three representative models covering the entire range of all predicted cyt c_2 binding positions. **h-j.** The SC is shown fitted inside the transparent low resolution cryo-EM map SC-2B (**Supplementary Fig. 5g**), subunits are colored as before. Twenty representative models of cyt domain of c_y docked onto $CIII_2$ monomer A are shown in different colors. **h** and **i** represent the back and front views (as in **Fig. 2a** and **d**) of the SC. A region exhibiting an extra feature at the SC interface is indicated by an arrow. **j** shows a top view to better visualize the two partially overlapping binding clusters of cyt domain of c_y, with one on cyt c_1 (arrow 1) and the other one at the interface of cyt c_1 and the FeS-ED protein (arrow 2) of $CIII_2$.

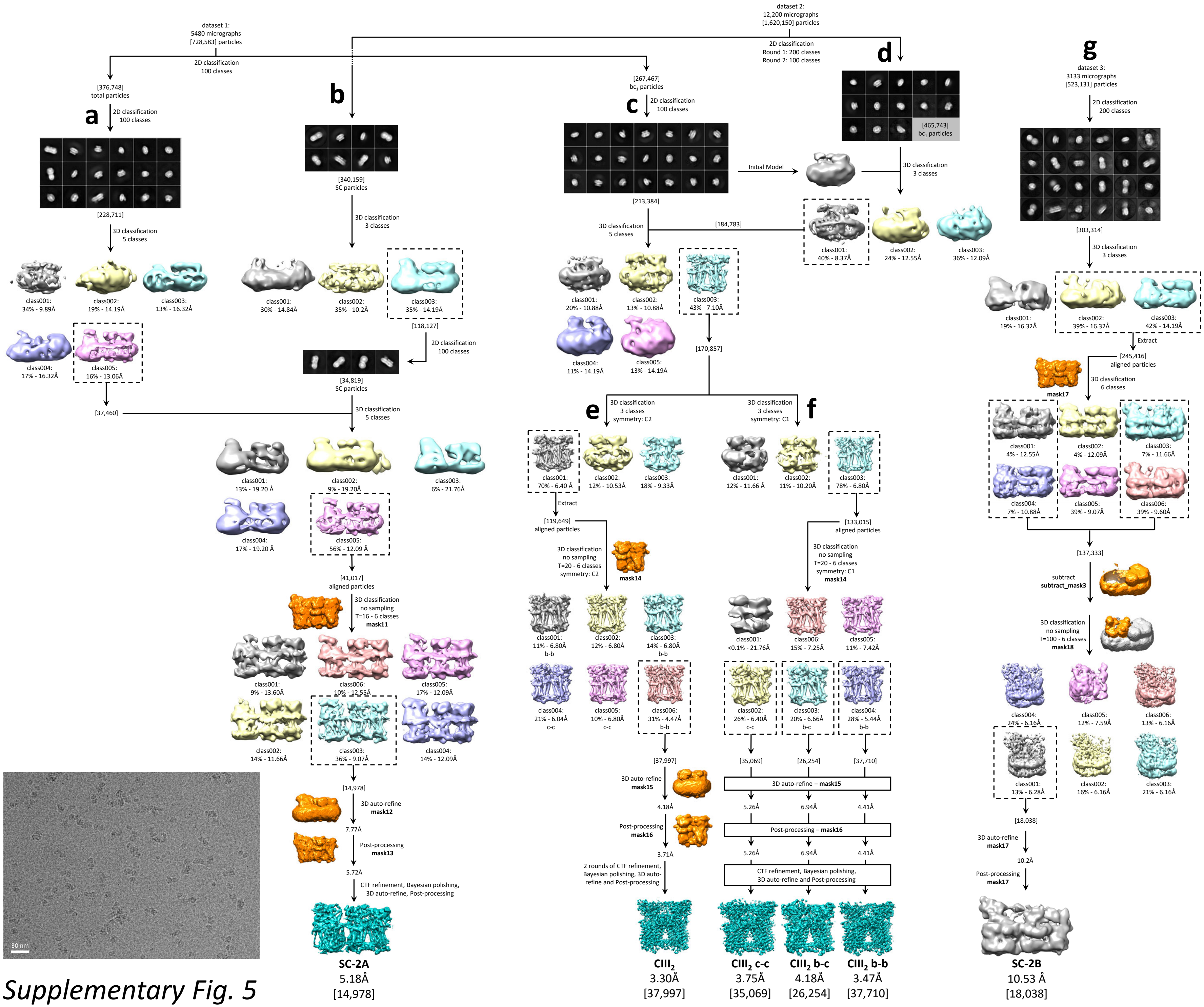

Supplementary Fig. 5

Supplementary Figure 5. Classification tree for bipartite SCs and $CIII_2$ conformers. A representative micrograph (out of 21,173) is shown at the bottom left corner with a white 30 nm scale bar. Three datasets were collected and particles were processed by Relion 3.0. Subclasses obtained in 3D classifications are shown in various colors, and labeled with class distribution (in %) and the nominal resolution as in **Supplementary Fig. 2**. All masks are shown in orange, and the final maps that are deposited to EMDB in turquoise. **a.** An initial 2D classification of particles from dataset-1 yielded 376,748 particles in suitable class averages, which were subjected to a second round of 2D classification. Class-averages likely representing different orientations of bipartite SCs were classified into five 3D classes using the same initial model used for the tripartite SC analyses (**Supplementary Fig. 2**), and a single class showing the entire SC at the highest resolution was selected. These particles were processed separately (not shown) as well as together with the SC particles from dataset-2 (**b**). **b.** After two rounds of 2D classification of particles from dataset-2, those likely representing SCs in different orientations were retained, and classified into three 3D classes using the same initial model as in **a**. The class most closely resembling the overall shape of bipartite $CIII_2CIV$ was chosen, and subjected to another round of 2D classification. Only those particles that clearly represent the SCs were retained and combined with the particles from dataset-1 (**a**). Combined SC particles from both datasets were classified into five 3D classes, the best of which was selected and further processed using a solvent mask. After 3D auto-refinement and post processing, a map containing 14,978 particles was obtained with a nominal resolution of 5.72 Å, which after per-particle CTF-refinement and Bayesian polishing led to map SC-2A at 5.18 Å resolution. **c.** Only the class averages resembling a $CIII_2$ dimer (without any bound CIV) were selected after the first round of 2D classification, and subjected to a second round of 2D classification after which appropriate class averages were selected, and a reference-free initial model was created in Relion 3.0. **d.** After two rounds of 2D

classification of particles from dataset-2, those resembling a $CIII_2$ dimer were selected and classified into three 3D classes using the initial model created in **c**. The class with the highest resolution and level of detail was selected and combined with the $CIII_2$ particles obtained in **c**. The combined $CIII_2$ particles were subjected to 3D classification into 5 classes and the best class was further processed, either imposing C2 symmetry (**e**) or assuming no symmetry (**f**). **e.** The class with the highest level of detail was selected and aligned particles were subjected to a second round of 3D classification without image alignment, using a solvent mask, and imposing C2 symmetry. Six classes were obtained, some of which showed both Rieske FeS protein external domain (FeS-ED) proteins in b, while other classes showed both FeS-ED proteins in c, positions. The class with the largest number of particles and the highest resolution was selected and subjected to several rounds of refinement, post processing, per-particle CTF-refinement and Bayesian polishing. The final map consisted of ~38,000 particles has a nominal resolution of 3.3 Å, and shows both FeS-ED proteins in b position. **f.** The same strategy as in (**e**) was used, but no symmetry (*i.e.*, C1 symmetry) was applied in the two rounds of 3D classification. As a consequence, besides both FeS-EDs being in the same conformation (b-b and c-c), a third state was observed with one FeS-ED in b, and the other in c position (b-c). This class representing the b-c conformational state was refined without imposing C2 symmetry, while the best classes representing the b-b and c-c conformational states were refined with C2 symmetry as in (**e**). Three distinct maps, representing the different conformations of FeS-ED proteins in $CIII_2$ were obtained. **g.** Upon analysis of a third dataset, some maps showed an additional feature at the interface of CIII and CIV, which was tentatively attributed to cyt *c* domain of c_y. To obtain the best map containing the extra density, all classes showing extra density at the interface were combined after the second round of 3D classification using a soft mask and the $CIII_2$ portion of the particles was subtracted. The remaining particles were sorted into 6 classes in another round of 3D

classification, using a mask that included CIV and the interface region. Resulting classes were subjected to 3D auto-refinement and post-processing, and the final map (SC-2B) showing the strongest density at the SC interface is shown.

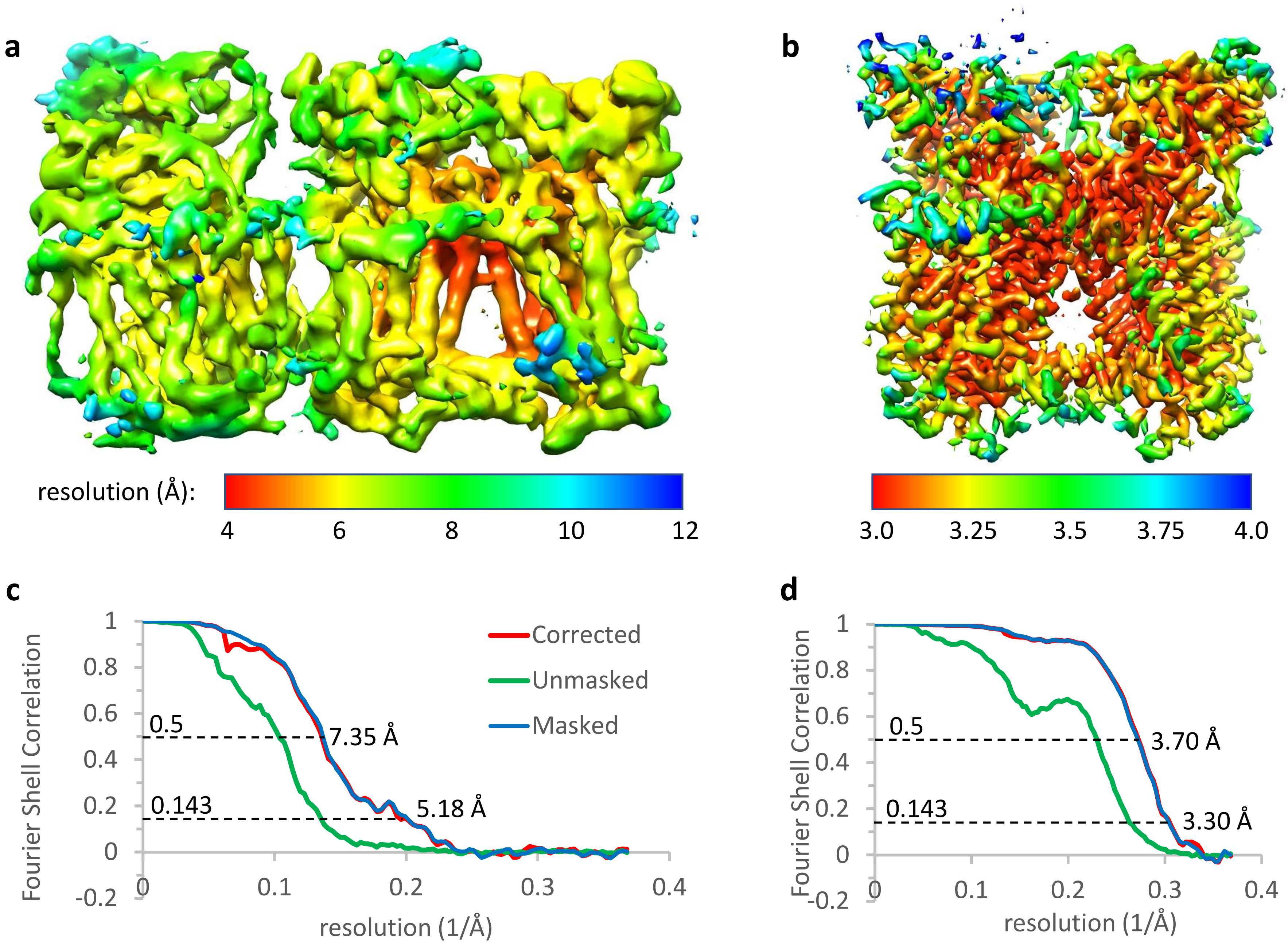

Supplementary Fig. 6

Supplementary Figure 6. Local resolutions of the cryo-EM maps SC-2A and CIII$_2$. a and **b**, local resolutions of SC-2A and CIII$_2$ maps, respectively. Local resolution was calculated in cryoSPARC [12], and the resolution range is shown at the bottom of each map. **c** and **d**, Fourier Shell Correlations (FSC) for SC-2A and CIII$_2$ maps, respectively. The FSC plots for the unmasked (green), masked (blue), and corrected (red) maps are shown, and the nominal resolution estimates for the corrected maps at FSC = 0.143 and 0.5 are indicated.

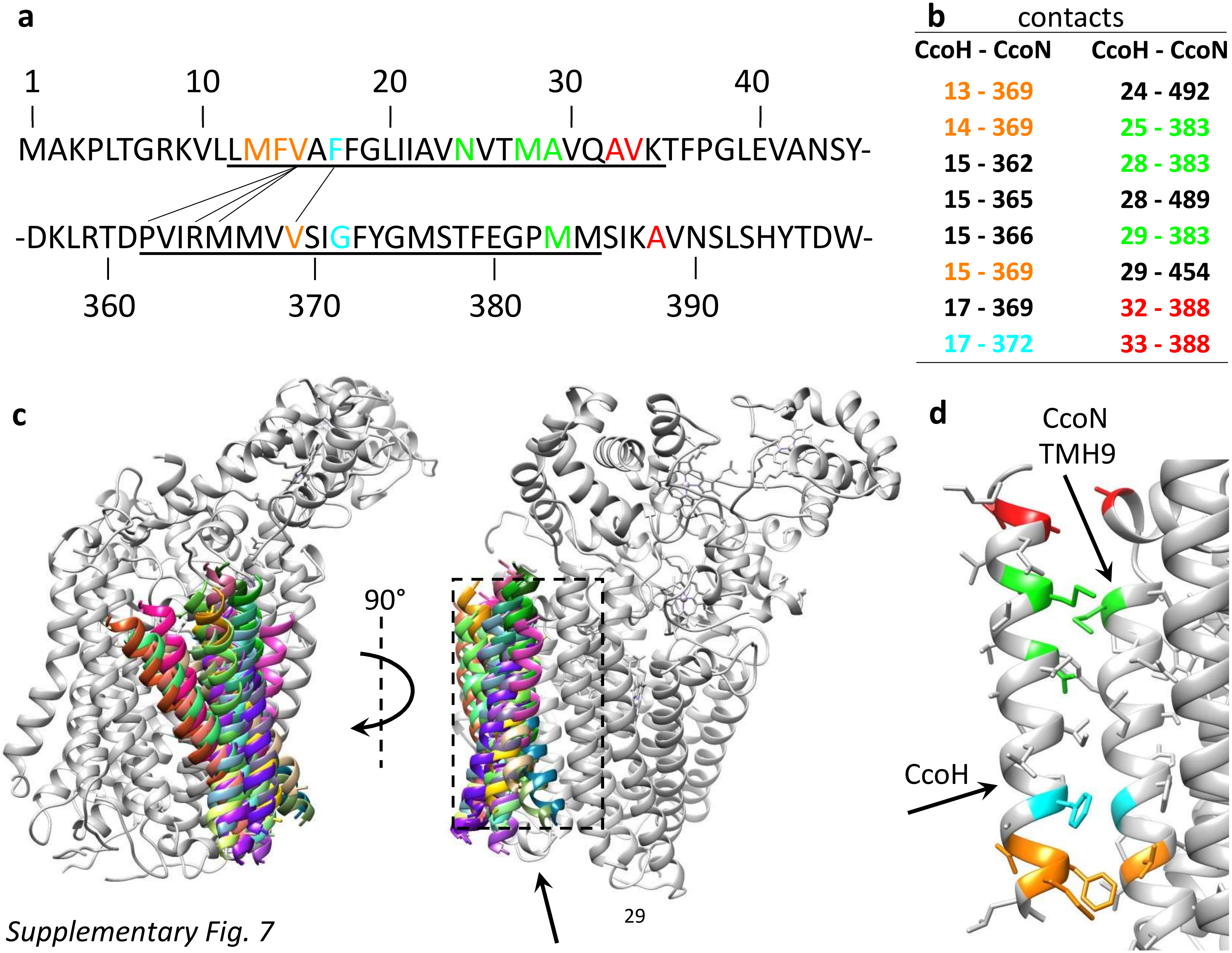

Supplementary Fig. 7

Supplementary Figure 7. Docking of CcoH to *cbb*3-type CIV. a. The first 45 N-ter amino acid residues of *R. capsulatus* CcoH, with its predicted TMH (residues 12 to 34, underlined), and the sequence of CcoN TMH9 are shown. The residues predicted to interact are indicated by the same colors, additional interactions partially overlapping with the color-coded ones are indicated by connecting lines. **b.** List of the predicted contacts by RaptorX-ComplexContact [13] between CcoH and CcoN residues, with interacting residues highlighted in their respective colors as in **a**. One contact between CcoH-115 and CcoN-386 was omitted because it is outside the modelled TMH region of CcoH. **c.** An *ab initio* model for this TMH portion of CcoH, generated by I-TASSER server [14], was docked to *cbb*3-type CIV using Patchdock [15] guided with the distance restraints provided by RaptorX-ComplexContact analysis. A cluster of 25 representative orientations of CcoH models (depicted in different colors) is shown on *R. capsulatus cbb*3-type CIV, where the CcoN, CcoO and CcoP subunits are colored gray. Views from two different angles are presented to better depict the location of CcoH TMH, near CcoN TMH9 (not labeled). **d.** Enlarged view of the region highlighted with a dashed box and arrow in (**c**), showing predicted interactions between CcoH TMH and CcoN TMH9. CcoH TMH is shown in its final position as modelled in map SC-2A (EMD-22227; PDB: 6XKW), cyt c_y and other TMHs of CIV are not shown. The entire structure is in grey, and the residues with predicted interactions are colored as in **a**. Some overlapping interactions listed in **b** are omitted for clarity.

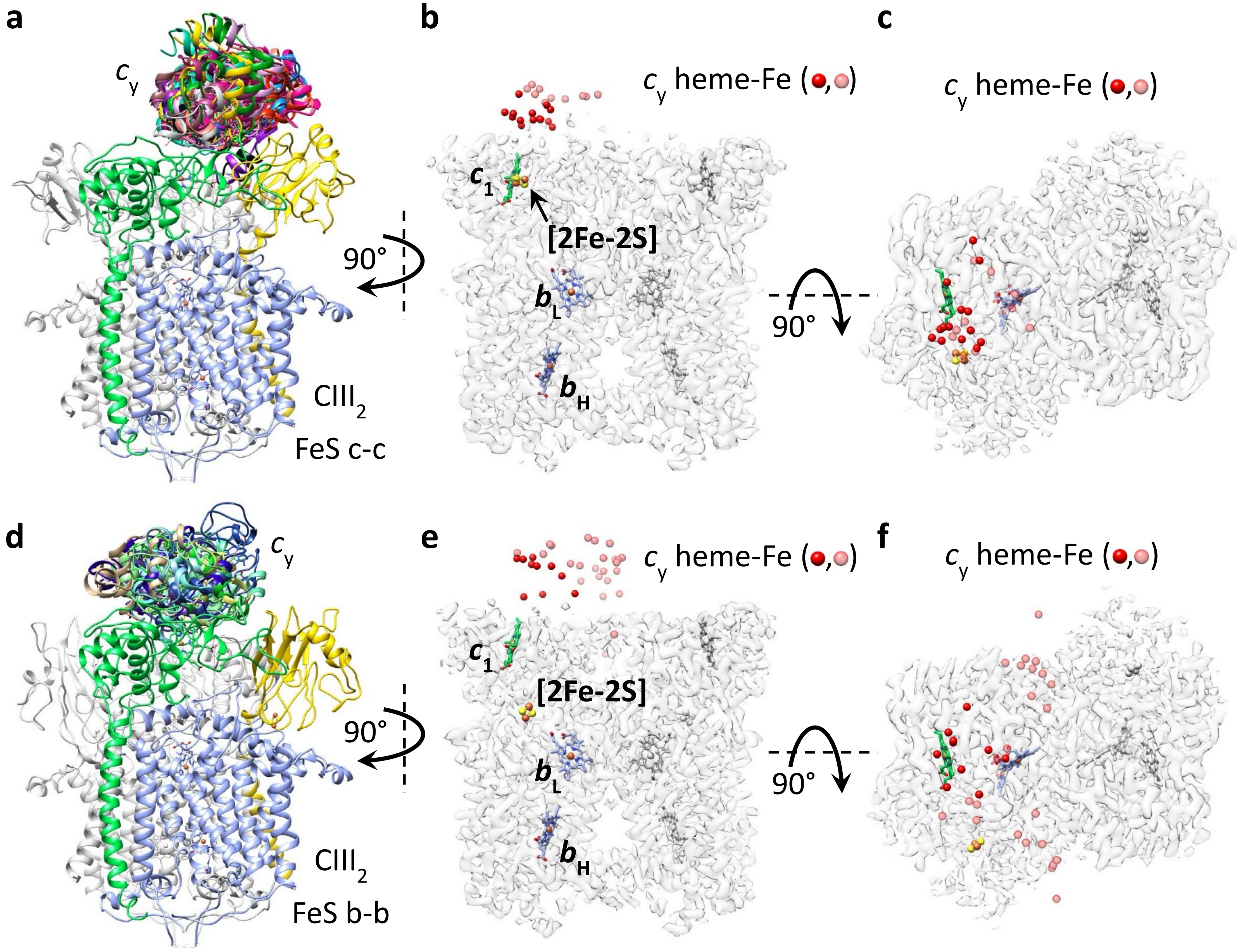

Supplementary Fig. 8

Supplementary Figure 8. Positions of the FeS-ED proteins affect docking of cyt *c* domain of c_y on $CIII_2$. Top and bottom rows show the XLs guided docking of cyt *c* domain of c_y onto two $CIII_2$ conformers with their FeS-ED proteins in c ($CIII_2$ c-c) and in b ($CIII_2$ b-b) positions, respectively. **a.** One major docking cluster was observed per monomer of $CIII_2$ c-c, and sixteen models of cyt *c* domain of c_y located on monomer A are shown. The subunits of monomer A are colored as in **Fig. 2** and those in monomer B shown in gray. **b** and **c** are the side and top views, respectively, of the semi-transparent map $CIII_2$-cc with its cofactors. The view shown here is similar to that of the SC in **Fig. 5a**, **c**. For clarity, the docking models of cyt *c* domain of c_y are represented by their heme-Fe atoms (spheres). Unlike **a**, which shows only the clustered models, the Fe atoms of all models (28 total) docked on monomer A are shown either by fully colored red spheres for those located within 25 Å, or by semi-transparent spheres for those beyond 25 Å of cyt c_1 heme-Fe. **d**. Two major docking clusters were observed per monomer of $CIII_2$ b-b, with one located on top of cyt c_1 (arrow 1 in **Supplementary Fig. 4j**) and the other one at the inter-monomer interface between cyt c_1 and the FeS-ED protein of the other monomer (arrow 2 in **Supplementary Fig. 4j**). Ten models of cyt *c* domain of c_y representing the cluster located on top of cyt c_1 in monomer A are shown here. **e** and **f** are the side and top views, respectively, of the map $CIII_2$-bb with its cofactors and the heme-Fe atoms of docked models of cyt *c* domain of c_y (33 total) on monomer A, as in **b** and **c**. Comparison of the top and bottom rows shows that the docking models of cyt *c* domain of c_y are clustered more tightly on top of cyt c_1 when the FeS-ED proteins of $CIII_2$ are in c position.

REFERENCES

1 Sambrook, J. & Russell, D. W. *Molecular Cloning: a laboratory manual*. 3rd ed. (Cold Spring Harbor Laboratory Press, 2001).

2 Zannoni, D., Prince, R. C., Dutton, P. L. & Marrs, B. L. Isolation and Characterization of a Cytochrome c_2-Deficient Mutant of *Rhodopseudomonas-capsulata*. *Febs Lett* **113**, 289-293, doi:Doi 10.1016/0014-5793(80)80611-9 (1980).

3 Koch, H. G., Hwang, O. & Daldal, F. Isolation and characterization of *Rhodobacter capsulatus* mutants affected in cytochrome cbb_3 oxidase activity. *J Bacteriol* **180**, 969-978 (1998).

4 Daldal, F. Cytochrome c_2-independent respiratory growth of *Rhodobacter capsulatus*. *J Bacteriol* **170**, 2388-2391, doi:10.1128/jb.170.5.2388-2391.1988 (1988).

5 Ozturk, Y. *et al.* Soluble variants of *Rhodobacter capsulatus* membrane-anchored cytochrome c_y are efficient photosynthetic electron carriers. *J Biol Chem* **283**, 13964-13972, doi:10.1074/jbc.M800090200 (2008).

6 Ditta, G. *et al.* Plasmids related to the broad host range vector, pRK290, useful for gene cloning and for monitoring gene expression. *Plasmid* **13**, 149-153, doi:10.1016/0147-619x(85)90068-x (1985).

7 Gray, K. A. *et al. Rhodobacter capsulatus* contains a novel *cb*-type cytochrome *c* oxidase without a Cu_A center. *Biochemistry* **33**, 3120-3127, doi:10.1021/bi00176a047 (1994).

8 Selamoglu, N. *et al.* Comparative differential cuproproteomes of *Rhodobacter capsulatus* reveal novel copper homeostasis related proteins. *Metallomics* **12**, 572-591, doi:10.1039/c9mt00314b (2020).

9 Gotze, M. *et al.* Automated assignment of MS/MS cleavable cross-links in protein 3D-structure analysis. *J Am Soc Mass Spectrom* **26**, 83-97, doi:10.1007/s13361-014-1001-1 (2015).

10 Kalisman, N., Adams, C. M. & Levitt, M. Subunit order of eukaryotic TRiC/CCT chaperonin by cross-linking, mass spectrometry, and combinatorial homology modeling. *Proc Natl Acad Sci U S A* **109**, 2884-2889, doi:10.1073/pnas.1119472109 (2012).

11 Poornam, G. P., Matsumoto, A., Ishida, H. & Hayward, S. A method for the analysis of domain movements in large biomolecular complexes. *Proteins* **76**, 201-212, doi:10.1002/prot.22339 (2009).

12 Punjani, A., Rubinstein, J. L., Fleet, D. J. & Brubaker, M. A. cryoSPARC: algorithms for rapid unsupervised cryo-EM structure determination. *Nat Methods* **14**, 290-296, doi:10.1038/nmeth.4169 (2017).

13 Zeng, H. *et al.* ComplexContact: a web server for inter-protein contact prediction using deep learning. *Nucleic Acids Res* **46**, W432-W437, doi:10.1093/nar/gky420 (2018).

14 Zhang, W. *et al.* Integration of QUARK and I-TASSER for Ab Initio Protein Structure Prediction in CASP11. *Proteins* **84 Suppl 1**, 76-86, doi:10.1002/prot.24930 (2016).

15 Schneidman-Duhovny, D., Inbar, Y., Nussinov, R. & Wolfson, H. J. PatchDock and SymmDock: servers for rigid and symmetric docking. *Nucleic Acids Res* **33**, W363-367, doi:10.1093/nar/gki481 (2005).
